# Can integrating the Memory Support Intervention into cognitive therapy improve depression outcome? Study protocol for a randomized controlled trial

**DOI:** 10.1186/s13063-017-2276-x

**Published:** 2017-11-14

**Authors:** Allison G. Harvey, Lu Dong, Jason Y. Lee, Nicole B. Gumport, Steven D. Hollon, Sophia Rabe-Hesketh, Kerrie Hein, Kirsten Haman, Mary E. McNamara, Claire Weaver, Armando Martinez, Haruka Notsu, Garret Zieve, Courtney C. Armstrong

**Affiliations:** 1 Department of Psychology, University of California, Berkeley, 3210 Tolman Hall #1650, Berkeley, CA 94720-1650 USA; 2 Vanderbilt University, Nashville, TN USA

**Keywords:** Memory support, Depression, Cognitive therapy, Transdiagnostic, Experimental therapeutics

## Abstract

**Background:**

The Memory Support Intervention was developed in response to evidence showing that: (1) patient memory for treatment is poor, (2) poor memory for treatment is associated with poorer adherence and poorer outcome, (3) the impact of memory impairment can be minimized by the use of memory support strategies and (4) improved memory for treatment improves outcome. The aim of this study protocol is to conduct a confirmatory efficacy trial to test whether the Memory Support Intervention improves illness course and functional outcomes. As a “platform” for the next step in investigating this approach, we focus on major depressive disorder (MDD) and cognitive therapy (CT).

**Method/design:**

Adults with MDD (*n* = 178, including 20% for potential attrition) will be randomly allocated to CT + Memory Support or CT-as-usual and will be assessed at baseline, post treatment and at 6 and 12 months’ follow-up (6FU and 12FU). We will compare the effects of CT + Memory Support vs. CT-as-usual to determine if the new intervention improves the course of illness and reduces functional impairment (aim 1). We will determine if patient memory for treatment mediates the relationship between treatment condition and outcome (aim 2). We will evaluate if previously reported poor treatment response subgroups moderate target engagement (aim 3).

**Discussion:**

The Memory Support Intervention has been developed to be “transdiagnostic” (relevant to a broad range of mental disorders) and “pantreatment” (relevant to a broad range of types of treatment). This study protocol describes a “next step” in the treatment development process by testing the Memory Support Intervention for major depressive disorder (MDD) and cognitive therapy (CT). If the results are promising, future directions will test the applicability to other kinds of interventions and disorders and in other settings.

**Trial registration:**

ClinicalTrials.gov, ID: NCT01790919. Registered on 6 October 2016.

**Electronic supplementary material:**

The online version of this article (doi:10.1186/s13063-017-2276-x) contains supplementary material, which is available to authorized users.

## Background

Patients accurately recall only about one third of the recommendations made during a physician visit [1–8] and during a cognitive behavior therapy (CBT) session [9, 10]. In one study, 25% of patients remembered recommendations that were *not* made [1]. Recall is particularly poor for advice for health-behavior change [11]. Perhaps not surprisingly, poor memory for the content of treatment is associated with poor adherence to medical treatments for chronic conditions [12–16], which is known to be associated with worse outcome [17]. Also, poor memory for CBT is associated with poorer outcome [10].

We have offered various explanations for these findings [18]. First, even when memory is functioning optimally, fallibility is possible at initial encoding, storage or retrieval [19]. Second, a CBT treatment session is typically 50 min long, covers complex information, and can elicit negative emotion. Negative emotion is associated with attentional biasing and narrowing, which impacts encoding [20]. Third, the odds are stacked *against* people learning, generalizing and transferring knowledge to new situations; this is known as the transfer of learning problem [21, 22]. Fourth, memory deficits and biases are common across mental disorders [23–27] and memory deficits are associated with poorer memory for treatment [28, 29].

The good news is that the impact of memory impairment can be minimized. Memory encoding and retention can be markedly improved by the use of memory support techniques. This has been demonstrated in medical visits [13, 30], and among older adults [31] as well as for people with memory impairments associated with Alzheimer’s disease, vascular dementia [32] and frontal lobe dysfunction [33].

We developed and tested the Memory Support Intervention, which is designed to improve patient memory *for treatment*. This approach is *not* intended to have a direct effect on improving memory functioning per se. The Memory Support Intervention was distilled from the cognitive science and education literature based on carefully honed criteria [18]. A small underpowered trial has provided preliminary evidence that the intervention exerts a measurable effect on patient memory for treatment and demonstrates a clinical effect [34].

The Memory Support Intervention has been developed to be “transdiagnostic” (relevant to a broad range of mental disorders) and “pantreatment” (relevant to a broad range of types of treatment). However, investigating these possible broader applications seems premature without first establishing efficacy, which is the aim of the study described in this paper. To create a platform for the “next step” in investigating the approach, we will recruit patients who meet diagnostic criteria for major depressive disorder (MDD) and focus on one biopsychosocial intervention—cognitive therapy (CT). If the results are promising, future directions will test the applicability to other kinds of interventions and disorders and in other settings.

Why focus on MDD? First, MDD is prevalent and causes impairment [35, 36]; second, there is a need to improve treatments for MDD because a proportion of patients do not recover and of those who do recover, the majority relapse [37]; third, memory is poor in MDD and is modifiable [38, 39]; fourth, preliminary data suggest that improving memory for treatment in MDD improves outcome [34, 40, 41].

Why focus on CT for MDD? CT for MDD is well articulated and has been widely studied. Meta-analyses confirm CT as a frontline treatment [42, 43]. Despite these impressive outcomes, there is room for improvement [44, 45].

This study protocol addresses two additional considerations. First, older age, lower intelligence and chronic depression each predict poorer response to treatment for depression, including CT [46]. Impairment in declarative memory is also associated with poorer outcome [47–50]. Moreover, fewer years of education has been associated with a positive response to CT + Memory Support relative to more years of education [34]. We hypothesize that all of these poorer response groups may derive special benefit from the Memory Support Intervention. Hence, while we expect all patients to benefit from the Memory Support Intervention, we have planned analyses to determine if these subgroups stand to gain the greatest advantage from experimental facilitation of memory for treatment. Second, there is evidence that improved mood in MDD is associated with improved declarative memory [51], although remitted MDD patients still exhibit significant memory deficits [23]. We have planned analyses to address this potential confound.

Over a 4-year period, adults with MDD (*n* = 178, including 20% for attrition) will be randomly allocated to CT + Memory Support or CT-as-usual and will be assessed at baseline, post treatment and at 6 and 12 months’ follow-up (6FU and 12FU). The first aim is to assess whether the Memory Support Intervention (1) improves the course of illness and (2) reduces functional impairment. Compared to those receiving CT-as-usual, we expect that patients receiving CT + Memory Support will experience greater symptom reduction on measures of course of illness, as defined by the American College of Neuropsychopharmacology (ACNP) Task Force criteria, and on measures of functional impairment. The second aim is to evaluate whether patient memory for treatment mediates the relationship between treatment condition and outcome. Relative to CT-as-usual, we expect CT + Memory Support to be associated with better treatment outcome, and will be mediated by better patient memory for treatment, measured by the Patient Treatment Recall Task. The third aim is to evaluate if previously reported poor treatment response subgroups moderate target engagement. We expect that the relationship between memory support dose and outcome will be positive and stronger among those who are older, have lower IQ, have more chronic depression, have poorer baseline declarative memory performance and have fewer years of education.

## Method/design

### Study design and setting

**Fig. 1:**
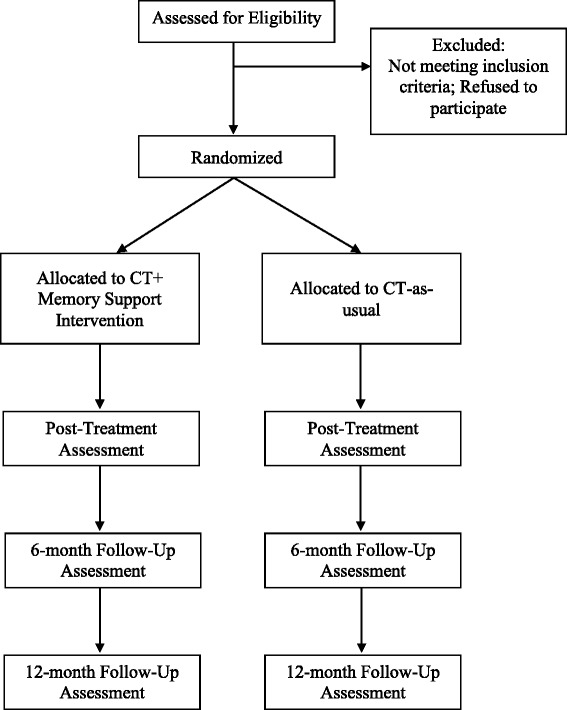
Standard Protocol Items: Anticipated patient flow for the randomized clinical trial

This is a prospective randomized controlled study. Adults (*n* = 178) who meet criteria for MDD will be randomly assigned, in a 1:1 parallel group design, to CT + Memory Support or CT-as-usual (see Fig. 1 for a flow chart of the study design). Randomization is stratified by age (≤49, 50 + years) and depression chronicity (< 2 years, ≥ 2 years) [46]. Participants receive a battery of outcome measures pre-treatment and post-treatment (i.e., within 2 weeks after the final treatment session) and at 6 and 12 months’ follow-up. Additional assessments of patient and therapist memory for treatment take place in weeks 4, 8, 12 and 16 of treatment for the mediation analysis.

**Fig. 2:**
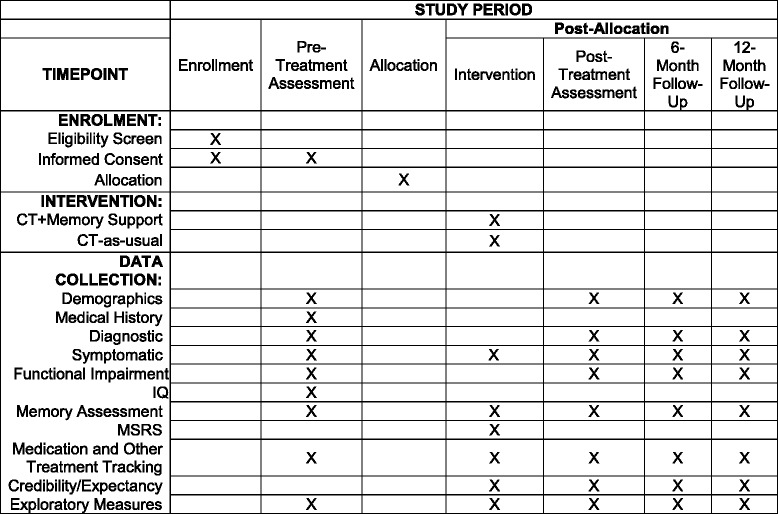
Standard Protocol Items: SPIRIT Figure: Schedule of enrollment, interventions, and assessments

The assessment team is blind to treatment allocation. Randomization will be conducted using a computerized, random-number generator where the planned stratified randomization is a part of the generation of the allocation sequence. Only the project coordinators in charge of randomization and of the Memory Support Rating Scale (MSRS) scoring know the treatment allocation of each participant. A Data Safety and Monitoring Board (DSMB) will review the study every 6 months during the active treatment phase. The Standard Protocol Items: Recommendations for Interventional Trials (SPIRIT 2013) Checklist (see Additional file 1) and Figure (see Fig. 2 Standard Protocol Item: Recommendation for Interventional Trials diagram) are provided [52].

For participants who discontinue, the assessment team will endeavor to collect all assessment data, prioritizing the primary outcomes.

### Participants

A total of 178 adults who meet the criteria will be recruited. Recruitment will be conducted in the Bay Area, CA, United States by clinician referral and advertisements. Eligibility is assessed first by a phone interview and then, if the individual is eligible after the phone interview, a more detailed, in-person interview. To enhance representativeness and generalizability, the inclusion and exclusion criteria are kept to a minimum.

### Inclusion criteria

The inclusion criteria are:

- Age 18 + years
- Willing and able to give consent. Participants must consent to being video-recorded (necessary for MSRS scoring) and to NIMH data sharing^1^
- English language fluency
- Diagnosis of major depressive disorder (MDD), first episode, recurrent or chronic according to the *Diagnostic and Statistical Manual of Mental Disorders, Fifth Edition* (DSM-5)
- Minimum score 26 or above on the Inventory of Depressive Symptomatology, Self-Report (IDS-SR). This cutoff denotes at least “moderate” depression
- If taking medications for mood, medications must be stable for the past 4 weeks

### Exclusion criteria

The exclusion criteria are:

- History of bipolar disorder
- History of psychosis or psychotic features
- Lifetime history of failure to respond to four or more sessions of CBT/CT for depression
- Current non-psychotic disorder if it constitutes the principal diagnosis and if it requires treatment other than that offered in the project. “Principal” is defined as the disorder currently most distressing and disabling, using a widely accepted severity rating scale capturing distress and interference (0–8, 4 + indicates clinical severity)
- Moderate or severe substance use in the past 6 months where “moderate” is defined as 4–5 symptoms and “severe” is defined as 6 + symptoms of those listed in DSM-5 for each of the substance-related disorders
- Evidence of any medical disorder or condition that could cause depression, preclude participation in CT, or is associated with memory problems, that is not currently stabilized and/or managed under the care of a physician or the presence of an active and progressive physical illness or neurological degenerative disease
- Current suicide risk sufficient to preclude treatment on an outpatient basis (assessed by the Columbia-Suicide Severity Rating Scale) or current homicide risk (assessed by our staff or referring treatment provider)
- Pregnancy or breastfeeding
- Not able/willing to participate in and/or complete the pre-treatment assessments

Excluding participants whose medications need to be changed is neither feasible nor representative of clinical practice [53]. Strategies to manage potential medication confounds are: (1) medications must be at stable doses for 4 weeks prior to randomization and (2) medication use and changes, along with participation in other treatments/therapy, will be recorded. All medication decisions will ultimately rest with the treating physician and participant.

Following the precedent set in prior research [54], participants with a lifetime history of failure to respond to four or more sessions of CBT for depression will be excluded. Participants who have engaged in moderate or severe substance use in the past 6 months will also be excluded. “Moderate” substance use is defined as four to five symptoms and “severe” substance use is defined as six or more of the symptoms listed in the DSM-5 for each of the substance-related disorders.

### Measures

The primary and secondary outcomes are presented in Additional file 2. Additional file 3 lists all of the measures as well as the timing for administration. Except where specified in Additional file 3, the measures described below will be delivered at each assessment point.

In addition to demographics (age, contact information, gender, race/ethnicity, family, education, employment, living arrangements, government assistance, housing) and assessment of medical history the following measures will be administered:

#### Diagnostic

To evaluate current and past psychiatric disorders the *Structured Clinical Interview for DSM-5 (SCID)* [55, 56] will be administered along with the *Longitudinal Interval Follow-up Evaluation (LIFE)* [57] to further ascertain presence/absence of MDD, number and length of mood episodes and remission, response, relapse, recovery, recurrence and time to relapse or recurrence (see Additional file 1 for definitions which are drawn from the ACNP criteria [58]).

#### Symptomatic

The *IDS-SR* [59], a widely used self-report measure of depression severity, as well as two subscales (ideation and behavior) from the lifetime and current version of the Columbia-Suicide Severity Rating Scale (C-SSRS) [60–62], will be administered. The *Quick Inventory for Depression Symptomatology* (QIDS) is administered every treatment session [38] for clinical purposes (monitoring of suicide and depression symptoms).

#### Functional impairment

The *World Health Organization Disability Assessment Schedule 2.0 (WHODAS 2.0)* [39] as well as the four-question *“Healthy Days”* (CDC HRQOL-4) core module developed by the CDC [63] will be administered.

#### IQ

The *National Adult Reading Test (NART)* [64] estimates premorbid intelligence. The total number of errors are calculated and used to derive an estimate of full-scale IQ and verbal IQ.

#### Memory

The *Patient Treatment Recall Task* [10] and the *Generalization Task* [65] are included as early indicators of target engagement and the trajectory of recall and learning over time. *Classical tests of memory* include a test of declarative memory—the *Episodic Face-Name Learning Task* [66–68]—which is the domain in which the most profound declines in MDD are observed [69–74]. Working memory will be assessed via the *N-Back* [75] because working memory aids the formation and retention of long-term declarative memories via attentive and elaborative-rehearsal processes [76, 77].

#### Memory Support Rating Scale (MSRS) [78]

Selected treatment session video-recordings will be coded using the *MSRS* to establish the dose of memory support delivered.

*Medication and other treatment tracking logs* will be used to record the additional treatments patients are receiving and any changes in those treatments throughout the duration of the study.

*Treatment credibility/expectancy* is administered at session 2, post-treatment, 6FU and 12FU via the *Credibility/Expectancy Questionnaire* [79, 80].

#### Exploratory measures

As this is a relatively new program of research and we wish to learn as much as possible, we have included the following measures on a pilot/exploratory basis: *Patient Usefulness and Utilization of Cognitive Therapy Skills Scale* (delivered post treatment, 6FU and 12FU), the *Competencies in Cognitive Therapy Scale* [81] (delivered at post treatment, 6FU and 12FU), *Patient Conceptualization of Depression Task* (delivered at the pre-treatment assessment, post treatment and 6FU) and a *Memory Support Treatment Provider Checklist* to be completed by the therapists delivering CT + Memory Support (delivered during weeks 4, 8, 12 and 16 of the treatment).

### Treatments

Both treatments are comprised of 20–26 × 50-min sessions conducted over 16 weeks.

#### CT-as-usual

CT was developed by Beck et al. [82] and has incorporated a number of innovations [83, 84]. Treatment maneuvers are designed to identify, reality test, and correct distorted beliefs and information processing [82]. CT for MDD will be conducted according to the standard manuals [82, 85, 86].

#### CT + Memory Support

The Memory Support Intervention is a manualized adjunctive treatment that will be delivered alongside CT-as-usual. The Memory Support Intervention is designed to improve patient memory for treatment. Distilled from the cognitive science and education literature based on carefully honed criteria [18], the Memory Support Intervention is comprised of eight memory-promoting strategies: attention recruitment, categorization, evaluation, application, repetition, practice remembering, cue-based reminder and praise recall. These strategies are proactively, strategically and intensively integrated into treatment-as-usual to support encoding. Memory support is delivered alongside each “treatment point,” defined as a main idea, principle, or experience that the treatment provider wants the patient to remember or implement as part of the treatment [10]. We acknowledge that some memory support is a standard part of certain treatments, including CBT [84, 87]. In the present study, the two treatment arms will differ in the “dose” of memory support. An underpowered pilot study indicated that the Memory Support Intervention effectively increases the amount of memory support delivered and improves depression outcome on certain measures [34].

#### Intervention fidelity

Treatment session video-recordings that occur at session 2 and at weeks 4, 8, 12 and 16 are coded using the MSRS [78] to establish the dose of memory support delivered. CT + Memory Support therapists are blind to which sessions are coded. CT-as-usual therapists are blind to the memory support portion of the research.

To reduce expectations impacting the delivery of each arm and to ensure purity of delivery [88], each treatment provider will be allocated to deliver only one of the two treatments. The rationale is that therapists report that once they have mastered memory support it is difficult to deliver CT-as-usual.

Clinicians use a treatment manual and receive weekly supervision to standardize treatment administration. Treatment sessions are video- and audio-taped and a random selection are rated using the *Cognitive Therapy Rating Scale* [89] which is a measure of general intervention skills and CBT-specific skills. The goal of this intervention is to maintain consistency and ensure adherence to the protocols [89].

### Data analysis

#### Preliminary data evaluation

Missing or aberrant data will be verified. Data will be audited for quality and completeness. Evaluation of distributions detects outliers and ensures that assumptions of planned analyses are met. Baseline differences between groups will be examined (e.g., IQ, demographics, psychiatric and medical comorbidity, medications). Statistical tests will not be used to select covariates in the primary intent-to-treat analysis [90–92]. Instead, the potential influences of baseline differences will be evaluated as moderators. If remedial training is given to a therapist whose adherence to the treatment protocol is not satisfactory, this will be included as a dummy-coded covariate in all analyses.

#### Multiple testing

We will use alpha = .05 for each primary hypothesis. For multiple testing conducted within each main hypothesis, we will adopt a stepwise multiple comparison procedure that is considered more powerful than the widely used Bonferroni correction [93, 94].

#### Missing data

Longitudinal analyses will use all available data and produce valid inferences if attrition depends on treatment group or on previous outcomes for the same participant [95]. If dropout is associated with other variables, they will be added as predictors to reduce bias due to data missing not at random.

#### Hypothesis 1

*CT + Memory Support will be superior to CT-as-usual for improving course of illness and reducing functional impairment at post-treatment, 6FU and 12FU on primary outcomes* listed in Additional file 1. Treatment groups will be compared on categorical outcomes (e.g., remission, relapse) using logistic regression to evaluate the odds of remission at post-treatment, and the odds of relapse at 6FU and 12FU. For the continuous variables (e.g., IDS-SR), we will test for differences in the mean trajectories across time between CT + Memory Support and CT-as-usual using Hierarchical Linear Modeling (HLM) [96–98]. The 1st level will represent within-person variation, and will include time indicators (or dummy variables) as predictors (post-treatment, 6FU and 12FU, with baseline as reference). The 2nd level represents between-person variation in the intercept and coefficients of the time indicators, and will include a dummy variable for arm (CT + Memory Support vs. CT-as-usual) as the predictor variable. Interactions between arm and the time indicators will be retained only if found to be significant at the 5% level using likelihood-ratio tests. Significant interaction terms between arm and time indicator suggest that there are different trajectories across time and between arms, and will be graphed to interpret the interaction.

#### Hypothesis 2

*Relative to CT-as-usual, the relationship between CT + Memory Support and better treatment outcome will be mediated by patient memory for treatment*. Outcome will be measured by IDS-SR at post-treatment, 6FU and 12FU. Patient memory will be measured by the Patient Treatment Recall Task and the Generalization Task at weeks 4, 8, 12 and 16 of treatment. A mediation model will be specified using Structural Equation Modeling [99]. Indirect effects from CT + Memory Support to better treatment outcome via patient memory will be assessed.

#### Hypothesis 3

The relationship between treatment condition and outcome will be moderated by poorer response subgroups; namely, older age, lower IQ, more chronic depression, poorer baseline declarative memory performance and fewer years of education. IQ will be estimated with the NART. Chronicity will be defined as current episode greater than or equal to 2 years [46]. Declarative memory will be quantified as described in Additional file 1. To assess whether Memory Support is more beneficial for any of the patient subgroups, moderation will be tested as an interaction between potential moderator and treatment arm; in the HLMs, the interaction between any of these variables, time, and treatment group will be tested.

#### Intervention fidelity check

MSRS scores will be compared for CT + Memory Support vs. CT-as-usual via independent samples *t* tests. To test the assumption that memory for CT session content improves as a result of CT + Memory Support we will use HLMs to analyze differences in linear rates of change between the two groups on the Patient Treatment Recall Task and Generalization Task delivered during weeks 4, 8, 12 and 16 of the treatment phase and at 6FU and 12FU.

### Additional planned analyses

1. *Is CT + Memory Support associated with longer time-to-relapse relative to CT-as-usual?* Survival analyses [100] will be conducted. The Kaplan-Meier product limit method will be used to generate survival curves for the two treatment conditions [101]. Cox regression will be applied to test for group differences.
2. *Are demographics, psychiatric and medical comorbidity, medications and symptom severity moderators?* This will be tested by adding the product of arm and the moderator variable to regression models or HLMs [102, 103]. A significant coefficient for the interaction term would indicate a moderating effect, and will be followed up with graphs to determine the nature of the effect modification [102].
3. *Does memory for treatment improve simply because memory deficits resolve with successful treatment?* Linear regressions will evaluate whether change in pre-post IDS and pre-post declarative and working memory each independently predict week 4 to post-treatment differences in Patient Recall Task performance. If significant, we will conduct a hierarchical linear regression to evaluate whether pre-post change in IDS scores predicts pre-post change in Patient Treatment Recall Task performance beyond pre-post improvement in memory.
4. *Memory Support Rating Scale* data will be used to delineate the most effective types and combinations of memory support. Factor analysis will be used to test the structure of the *Memory Support Rating Scale* based on Lee et al. [78]. Associations between patient recall, depression outcomes, and memory support variables will be assessed.
5. *Number of sessions received* (range 20–26 sessions) will be added as a covariate to control for differences in this across participants.

#### Power analysis

Based on the pilot study [34], using Glimmpse [104, 105] for repeated measures design, we took into account the four repeated measurements (pre-treatment, post-treatment, 6FU, 12FU). At an alpha level of 0.05 with 80% power, the sample size is estimated to be 80 for the IDS-SR. We also used G*Power and the Demidenko [106] method to calculate estimated sample size for binary mood outcomes (i.e., remission, relapse). The sample sizes are estimated to be 103 (remission) and 118 (relapse). Finally, Fritz and MacKinnon [107] provide recommendations for mediation sample size, and estimates from pilot data indicate that the recommended sample size is 148. Adding an additional 20% for possible attrition, we propose a sample of 178.

## Discussion

This study protocol addresses several research priorities. First, following the experimental therapeutics approach [108], this study protocol describes a confirmatory efficacy trial to provide a definitive test of the hypothesis that a novel target—patient memory for the contents of treatment—probed with a novel intervention—the Memory Support Intervention—will improve clinical outcome. Second, a novel intervention derived from basic, non-patient research in cognitive science and education, will be tested in the service of improving outcomes for patients with severe mental disorders. Third, the Memory Support Intervention has been derived to be “transdiagnostic” (relevant to a broad range of mental disorders) and “pantreatment” (relevant to a broad range of types of treatment). However, investigating these possible broader applications seems premature without first establishing efficacy, which is the aim of the study described in this protocol. If the results are promising, future directions will test the applicability to a broad range of biopsychosocial interventions for a wide range of patient populations being treated at various medical and mental health settings.

### Trial status

The trial is funded for 4 years (Project ID Number: R01MH108657). The research staff team started setting up the study in September, 2016. Patients were first randomized in December, 2016. The treatment phase will be completed by August of 2019. Final outcome assessments will be complete by August of 2020.

## Additional files

Additional file 1: SPIRIT 2013 Checklist. (DOC 122 kb)

Additional file 2: Summary of primary and secondary outcome(s). (DOCX 13 kb)

Additional file 3: Timeframe for Assessments. Table indicating time points where assessments are conducted. (DOCX 46 kb)

## Abbreviations

12FU: 12-months’ follow-up assessment
6FU: 6-months’ follow-up assessment
ACNP: American College of Neuropsychopharmacology
CBT: Cognitive behavior therapy
CEQ: Credibility/Expectancy Questionnaire
C-SSRS: Columbia-Suicide Severity Rating Scale
CT: Cognitive therapy
CTRS: Cognitive Therapy Rating Scale
DSM-5: *Diagnostic and Statistical Manual of Mental Disorders, Fifth Edition*
DSMB: Data and Safety Monitoring Board
HLM: Hierarchical Linear Modeling
IDS-SR: Inventory of Depressive Symptoms, Self-Report
LIFE: Longitudinal Interval Follow-up Evaluation
MDD: Major depressive disorder
MSRS: Memory Support Rating Scale
NART: National Adult Reading Test
NIMH: National Institutes of Mental Health
QIDS: Quick Inventory of Depressive Symptoms
SEM: Structural Equation Modeling
SPIRIT 2013: Standard Protocol Items: Recommendations for Interventional Trials
WHODAS 2.0: The World Health Organization Disability Assessment Schedule 2.0

## Footnotes

1 This was added in July, 2017. NIH/NIMH data-sharing requirements necessitated reconsent of participants already randomized. If a patient does not agree to data sharing we have been instructed to exclude them from the analysis.
