# Supplementary material for: Can integrating the Memory Support Intervention into cognitive therapy improve depression outcome? Study protocol for a randomized controlled trial
Source: Trials. 2017 Nov 14;18:539. doi: 10.1186/s13063-017-2276-x (PMC5686897; doi:10.1186/s13063-017-2276-x)
Supplement: Supplementary file 2 — Summary of primary and secondary outcome(s). (DOCX 13 kb) [file 13063_2017_2276_MOESM2_ESM.docx]

| Additional file 2. Summary of Primary and Secondary Outcome(s) | | |
| --- | --- | --- |
|  | **Primary outcome** | **Secondary outcome(s)** |
| Course of Illness: Pre-Post Treatment | % Remission is defined as signs and symptoms absent or close to it for ≥3 weeks and operationalized as IDS-SR ≤14 | a) % Response is defined as a clinically significant degree of symptom reduction and operationalized as 50% reduction in pre-treatment symptom severity on the IDS-SR  b) Magnitude of symptom change is operationalized as change on IDS-SR |
| Course of Illness: Post Treatment to 6 and 12 month FU | % Relapse is defined as ‘return to an MDE following remission’ and operationalized as ≥14 on the IDS-SR at follow-up for those who had remitted | a) % Recovery is defined as ‘remission sustained for (≥4 months’ and is operationalized via the SCID and LIFE.  b) % Recurrence is defined as ‘return to an MDE following recovery’ and is established using the SCID and LIFE  c) Time to relapse or recurrence following response or remission will be established using the SCID and LIFE.  d) Magnitude of symptom change will be established by the IDS-SR |
| Functional Impairment | WHODAS 2.0 total score | 4-question Healthy Days core module |
| Patient Memory for Treatment | Cumulative Recall on the Patient Treatment Recall Task | Past Session Recall on the Patient Treatment Recall Task |
| Generalization Task | Cognitive and behavioral generalization and number of accurate thoughts and applications | N/A |
| Declarative Memory | Hit Rate minus False Alarm Rate minus Lure Rate on the Episodic Face-Name Learning Task | N/A |
| Working Memory | No. of correct hits minus the no. of false positives on 3-Back of the N-Back | No. of correct hits minus the no. of false positives on 0-Back, 1-Back, 2-Back of the N-Back |
| Memory Support | Total Amount on the Memory Support Rating Scale | Number of Types on the Memory Support Rating Scale |
