# Supplementary material for: Can integrating the Memory Support Intervention into cognitive therapy improve depression outcome? Study protocol for a randomized controlled trial
Source: Trials. 2017 Nov 14;18:539. doi: 10.1186/s13063-017-2276-x (PMC5686897; doi:10.1186/s13063-017-2276-x)
Supplement: Supplementary file 3 — Timeframe for Assessments. Table indicating time points where assessments are conducted. (DOCX 46 kb) [file 13063_2017_2276_MOESM3_ESM.docx]

Additional file 3. Timeframe for Assessments

|  | Pre-Treatment | Treatment Weeks (20-26 sessions over 16 weeks) | | | | | | | | | | | | | | | | 2-Weeks Post-Treatment | 6-Month Follow-up | 12-Month Follow-up |
| --- | --- | --- | --- | --- | --- | --- | --- | --- | --- | --- | --- | --- | --- | --- | --- | --- | --- | --- | --- | --- |
|  |  | 1 | 2 | 3 | 4 | 5 | 6 | 7 | 8 | 9 | 10 | 11 | 12 | 13 | 14 | 15 | 16 |  |  |  |
|  |  |  |  |  |  |  |  |  |  |  |  |  |  |  |  |  |  |  |  |  |
| Telephone Screen | ● |  |  |  |  |  |  |  |  |  |  |  |  |  |  |  |  |  |  |  |
| Demographics | ● |  |  |  |  |  |  |  |  |  |  |  |  |  |  |  |  | ● | ● | ● |
| Medical History | ● |  |  |  |  |  |  |  |  |  |  |  |  |  |  |  |  |  |  |  |
| Structured Clinical Interview for DSM-5 (SCID) | ● |  |  |  |  |  |  |  |  |  |  |  |  |  |  |  |  | ● | ● | ● |
| Longitudinal Interval Follow-up Evaluation (LIFE) | ● |  |  |  |  |  |  |  |  |  |  |  |  |  |  |  |  | ● | ● | ● |
| Inventory for Depressive Symptomatology-Self-Report (IDS-SR) | ● |  |  |  | ● |  |  |  | ● |  |  |  | ● |  |  |  | ● | ● | ● | ● |
| Columbia-Suicide Severity Rating Scale (C-SSRS) | Administered on an ‘as needed’ basis as a safety screen for suicidality (i.e., C-SSRS is not an outcome measure) | | | | | | | | | | | | | | | | | | | |
| Quick Inventory for Depressive Symptomatology-Self-Report (QIDS) |  | ● | ● | ● | ● | ● | ● | ● | ● | ● | ● | ● | ● | ● | ● | ● | ● |  |  |  |
| WHODAS 2.0; Healthy Days | ● |  |  |  |  |  |  |  |  |  |  |  |  |  |  |  |  | ● | ● | ● |
| IQ (NART) | ● |  |  |  |  |  |  |  |  |  |  |  |  |  |  |  |  |  |  |  |
| Patient Treatment Recall Task |  |  |  |  | ● |  |  |  | ● |  |  |  | ● |  |  |  | ● |  | ● | ● |
| Generalization Task |  |  |  |  | ● |  |  |  | ● |  |  |  | ● |  |  |  | ● |  | ● | ● |
| Declarative memory and working memory tests | ● |  |  |  |  |  |  |  |  |  |  |  |  |  |  |  |  | ● | ● | ● |
| Memory Support Rating Scale (MSRS) |  | ● |  |  | ● |  |  |  | ● |  |  |  | ● |  |  |  | ● |  |  |  |
| Medication and Other Treatment Tracking | ● | ● | ● | ● | ● | ● | ● | ● | ● | ● | ● | ● | ● | ● | ● | ● | ● | ● | ● | ● |
| Credibility/Expectancy Questionnaire (CEQ) |  |  | ● |  |  |  |  |  |  |  |  |  |  |  |  |  |  | ● | ● | ● |
| Patient Usefulness and Utilization of CT Skills Scale |  |  |  |  |  |  |  |  |  |  |  |  |  |  |  |  |  | ● | ● | ● |
| Competencies of Cognitive Therapy Scale (CCTS-SR) |  |  |  |  |  |  |  |  |  |  |  |  |  |  |  |  |  | ● | ● | ● |
| Patient Conceptualization of Depression Task | ● |  |  |  |  |  |  |  |  |  |  |  |  |  |  |  |  | ● | ● |  |
| Memory Support Treatment Provider Checklist |  |  |  |  | ● |  |  |  | ● |  |  |  | ● |  |  |  | ● |  |  |  |
